# Supplementary material for: Mettl3-dependent m6A modification attenuates the brain stress response in Drosophila
Source: Nat Commun. 2022 Sep 14;13:5387. doi: 10.1038/s41467-022-33085-3 (PMC9474545; doi:10.1038/s41467-022-33085-3)
Supplement: Supplementary file 1 — Supplementary Information [file 41467_2022_33085_MOESM1_ESM.pdf]

Supplementary Materials for

Mettl3-dependent m^{6}A modification attenuates the brain stress response in *Drosophila*

Alexandra E. Perlegos[1], Emily J. Shields[2,3,4], Hui Shen[5], Kathy Fange Liu[5,6], Nancy M. Bonini[1,2*]

[6]Lead Contact
* Correspondence: nbonini@sas.upenn.edu (N.M.B)

[1]Neuroscience Graduate Group, University of Pennsylvania, Philadelphia, PA, 19104, USA.
[2]Department of Biology, University of Pennsylvania, Philadelphia, PA, 19104, USA.
[3]Epigenetics Institute and Department of Cell and Developmental Biology, University of Pennsylvania Perelman School of Medicine, Philadelphia, PA, USA
[4]Department of Urology and Institute of Neuropathology, Medical Center-University of Freiburg, Faculty of Medicine, University of Freiburg, Freiburg, Germany.
[5]Department of Biochemistry and Biophysics, Perelman School of Medicine, University of Pennsylvania, Philadelphia, PA, 19104, USA.
[6]Graduate Group in Biochemistry and Molecular Biophysics, Perelman School of Medicine, University of Pennsylvania, Philadelphia, PA, 19104, USA.

This PDF includes:

Supplementary figures 1-9

Other supporting materials include the following:

Supplementary Data 1: Full De-novo Motif files
Supplementary Data 2: m^{6}A Gene Lists.RADAR.
Supplementary Data 3: GO term and Kegg Pathway lists
Supplementary Data 4: S2 cell vs brain, head vs brain RNA-seq
Supplementary Data 5: Brain RNA-seq Differential expression lists
Supplementary Data 6: FlyBase HS gene list
Supplementary Data 7: *Drosophila* Lines and Primers used
Supplementary Data 8: Mapping rates for all sequencing experiments
Source Data file

Supplementary Figure 1 Validation of m^{6}A knockdown

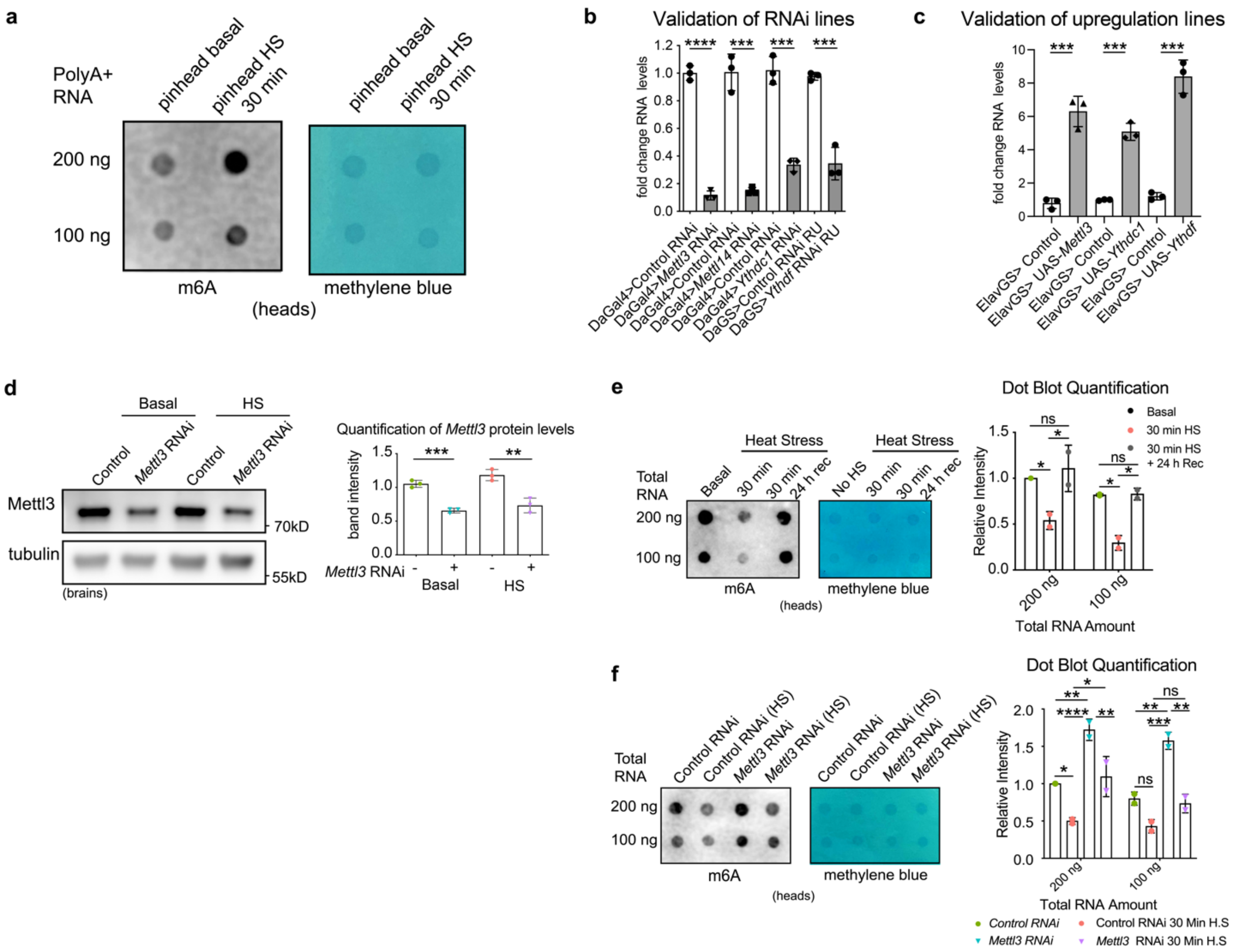

Supplementary Figure 1 Validation of m^{6}A knockdown

a Dot blot of m^{6}A on polyA+ RNA levels of basal and 30 min HS from heads of the *eya* allele *pinhead* (which is eyeless). These flies have reduced eye and head tissue. n =1 biological replicate, 200 heads per replicate per condition.

b RNA levels of m^{6}A pathway components (*Mettl3*, *Mettl14*, *Ythdf*) were assessed by qPCR to determine RNAi efficacy. DaGal4> mCherry RNAi; DaGal4>*Mettl3* RNAi; DaGal4>*Mettl14* RNAi, DaGal4>Ythc1 RNAi, DaGS> *Ythdf* RNAi. n=3 biological replicates, 15 heads per replicate. Data are presented as mean ± SD, ***p<0.001, ****p<0.0001, Student's two-tailed t-test. p<0.0001, p= 0.0004, p= 0.0004, p= 0.0004.

c RNA levels assessed by RT-qPCR to determine upregulation efficacy in brains of flies expressing UAS-*Mettl3*, UAS-*Ythdf*, UAS-*Ythdc1* in neurons. ElavGS> UAS-mCherry; ElavGS>UAS-*Mettl3,* ElavGS> UAS-*Ythdc,* ElavGS> UAS-*Ythdf*. n=3 biological replicates, 15 brains per replicate. Data are presented as mean ± SD, ***p<0.001, Student's two-tailed t-test. p= 0.0006, p= 0.0002, p= 0.0003.

d *Mettl3* protein levels from DaGal4> mCherry RNAi *or* DaGal4> *Mettl3* RNAi brains dissected in basal or HS 30 min at 38.5 °C. n= 3 biological replicates, 15 brains per replicate. Quantification of biological replicate immunoblots showing decreased expression of Mettl3 protein in *Mettl3* RNAi fly brain in basal and HS conditions. Data are presented as mean ± SD, **p<0.01, ***p<0.001, Student's two-tailed t-test. p= 0.0004, p= 0.0047.

e Dot blot of total RNA m^{6}A levels in fly heads (BL5905) extracted in basal, HS, or HS with recovery of 24 h. Quantification of 2 biological replicate blots of total RNA. m^{6}A levels that have been normalized to basal (intensity = 1) and methylene blue. n = 100 heads per condition per replicate. Data are presented as mean ± SD, *p-value<0.05, **p-value<0.01, two-way ANOVA. p=0.0476, p=0.018, p=0.0267, p=0.0245, ns = not significant.

f Dot blot analysis of total RNA m^{6}A levels in basal or HS conditions in DaGal4> mCherry RNAi or DaGal4> *Mettl3* RNAi. n = 2 biological replicate blots, 100 heads per condition. Data are presented as mean ± SD, *p-value<0.05, **p-value<0.01, ***p-value<0.001, ****p-value<0.001, two-way ANOVA. For 200ng, p=0.032, p=0.0036, p<0.0001, p=0.012, p=0.0088, for 100ng p=0.0023, p=0.0001, p=0.0013, ns= not significant.

Source data and statistical analysis are provided as a Source Data file.

Supplementary Figure 2 The brain has a distinct stress response

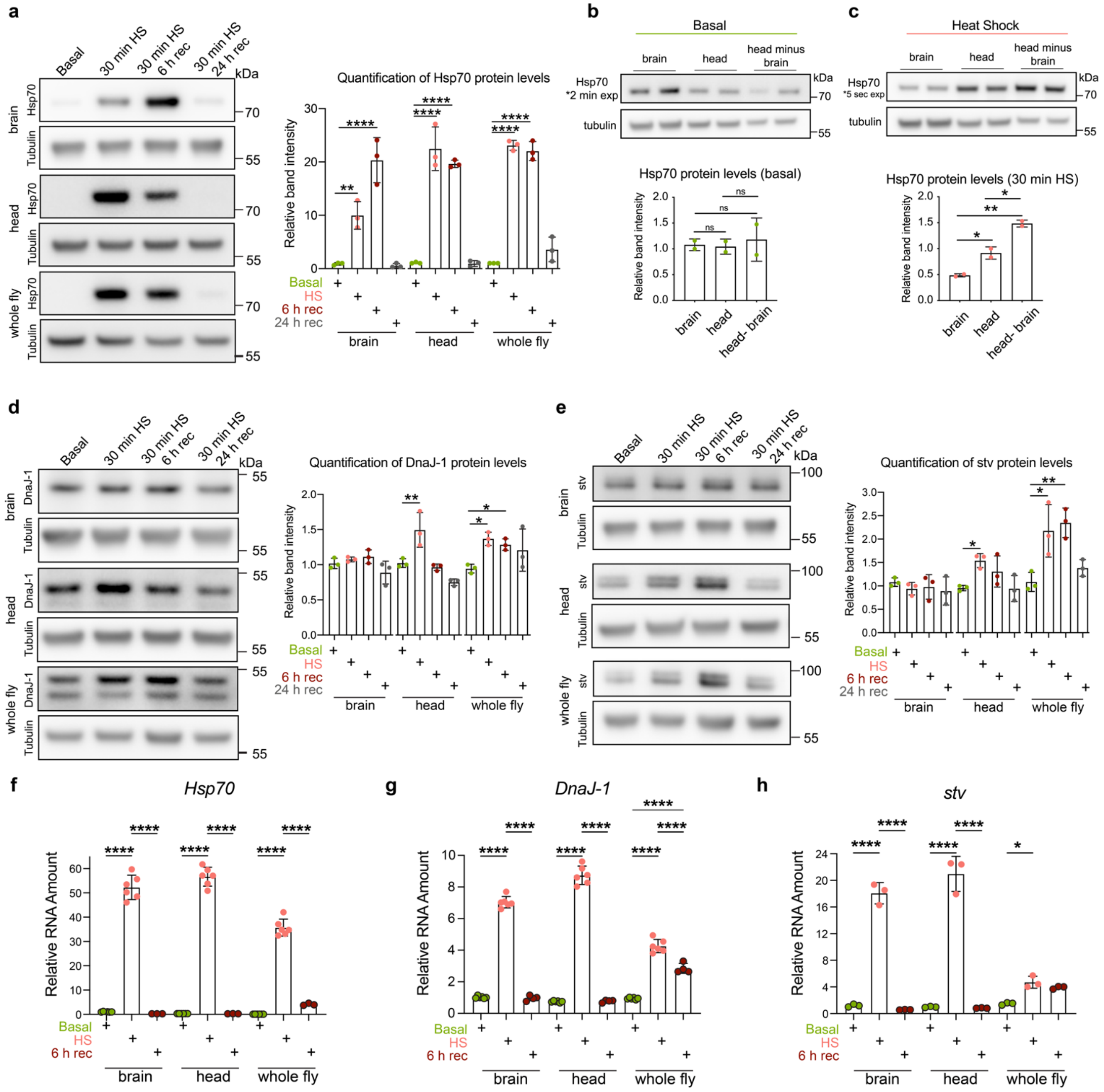

Supplementary Figure 2 The brain has a distinct stress response

a Time course analysis of Hsp70 protein levels in basal, HS 38.5 °C (HS) for 30 min, HS 30 min with 6 h recovery, HS 30 min with 24 h recovery from control fly (BL5905) brains, heads, and whole fly tissue. Tubulin used as the loading control. n=3 biological replicates per tissue type, 15 brains or heads per replicate, 6 whole flies per replicate. Data are presented as mean ± SD, **p<0.01; ****p<0.0001; one-way ANOVA with Dunnett's test across tissue type, compared to basal. For brain p=0.0053, p<0.0001, for head p<0.0001, p<0.0001, for whole fly p<0.0001, p<0.0001.

b Hsp70 protein levels in dissected brain, head, or head tissue with brain removed (outer cuticle) in basal conditions. No significant changes in Hsp70 protein per tissue type. n= 2 biological replicates, 15 flies per replicate. Data are presented as mean ± SD, one-way ANOVA with Tukey's test.

c Hsp70 protein levels in dissected brain, head, or head tissue with brain removed (outer cuticle) after HS 30 min. Significant increase in Hsp70 levels in heads and heads minus brains compared to brain tissue. n= 2 biological replicates, 15 flies per replicate. Data are presented as mean ± SD, *p<0.05; **p<0.01; one-way ANOVA with Tukey's test.

d-e Time course analysis of DnaJ-1 (HSP-40) and stv (BAG3) protein levels in basal, HS 30 min at 38.5 °C, HS 30 min and 6 h rec, HS 30 min and 24 h rec from brains, heads, and whole fly tissue. n=3 biological replicates per tissue type, 15 brains or heads per replicate, 6 whole flies per replicate. Data are presented as mean ± SD *p<0.05; **p<0.01; one-way ANOVA with Dunnett's test across tissue type. For DnaJ-1, for brain ns= not significant, for head p= 0.0059, for whole fly p= 0.0207. For stv, for brain ns= not significant, for head p= 0.0353, for whole fly p= 0.0126, p=0.0056.

f-h RT-qPCR analysis of *Hsp70*, *DnaJ-1*, and *stv* RNA levels from brains, heads, and whole fly tissue at basal, HS 30 min, HS 30 min + 6 h rec. RNA levels relative to basal no stress, normalized to b-tubulin. For *Hsp70* and *DnaJ-1* n=6 biological replicates per tissue type for control and HS conditions and n=4 biological replicates for 6 h rec, for *stv* n=3 biological replicates for all conditions. 15 brains or heads per replicate, 6 whole flies per replicate. Data are presented as mean ± SD, *p<0.05, ****p<0.0001; one-way ANOVA with Tukey's test across tissue type. For *Hsp70*, p<0.0001, for *DnaJ-1* p<0.0001, for *stv* p<0.0001, p= 0.0344.

Source data and statistical analysis are provided as a Source Data file.

Supplementary Figure 3 *Mettl3* knockdown increases heat stress resilience

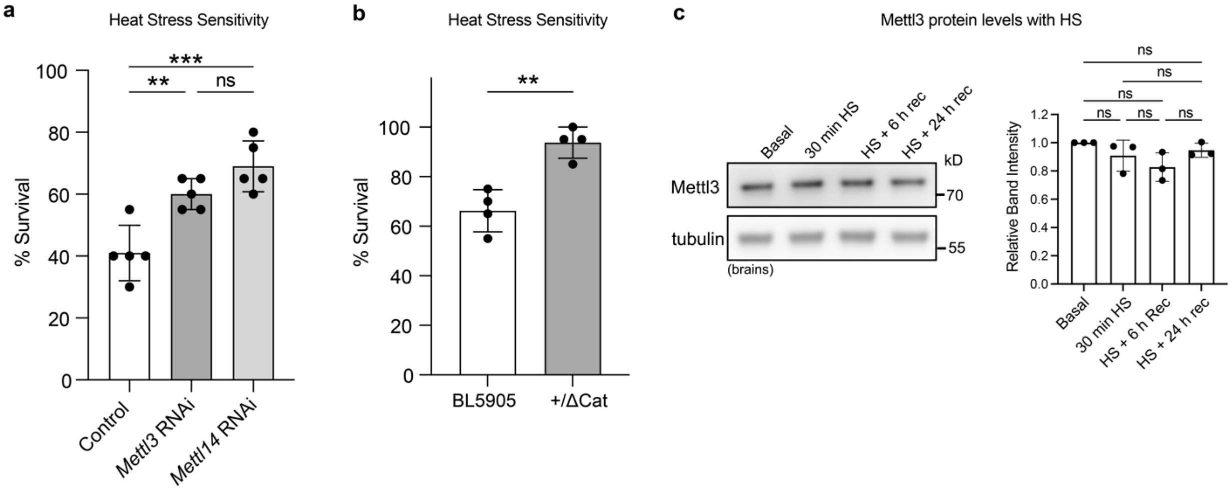

Supplementary Figure 3 *Mettl3* knockdown increases heat stress resilience

a Ubiquitous knockdown (DaGal4> *Mettl3* RNAi; DaGal4>*Mettl14* RNAi) and corresponding controls (mCherry RNAi) were HS for 1.5 hours at 38.5 °C and scored for survival after 24 hours recovery. n=5 biological replicates, each data point represents percent survival in vial of 20 flies per replicate. Data are presented as mean ± SD, **p<0.05, ***p<0.001, one-way ANOVA. p= 0.0004, p=0.0066, ns= not significant.

b *Mettl3* ΔCatalytic domain mutant crossed to BL5905 (ΔCat/+) or BL5905 (+/+) were HS for 1.5 hour at 38.5 °C and scored for survival after 24 h recovery. n=4 biological replicates, each data point represents percent survival in vial of 20 flies per replicate. Data are presented as mean ± SD, **p<0.05, student's t-test. p= 0.002.

c Mettl3 protein levels from control BL5905 brains dissected in basal, HS 30 min at 38.5 °C, HS + 6 h recovery, or HS + 24 h recovery. n= 3 biological replicates, 15 brains per replicate. Quantification of biological replicate immunoblots showed no significant change in Mettl3 protein levels with HS. Data are presented as mean ± SD, one-way ANOVA, ns=not significant.

Source data and statistical analysis are provided as a Source Data file.

Supplementary Figure 4 m^6A-IP seq using two antibodies (NEB and SYS).

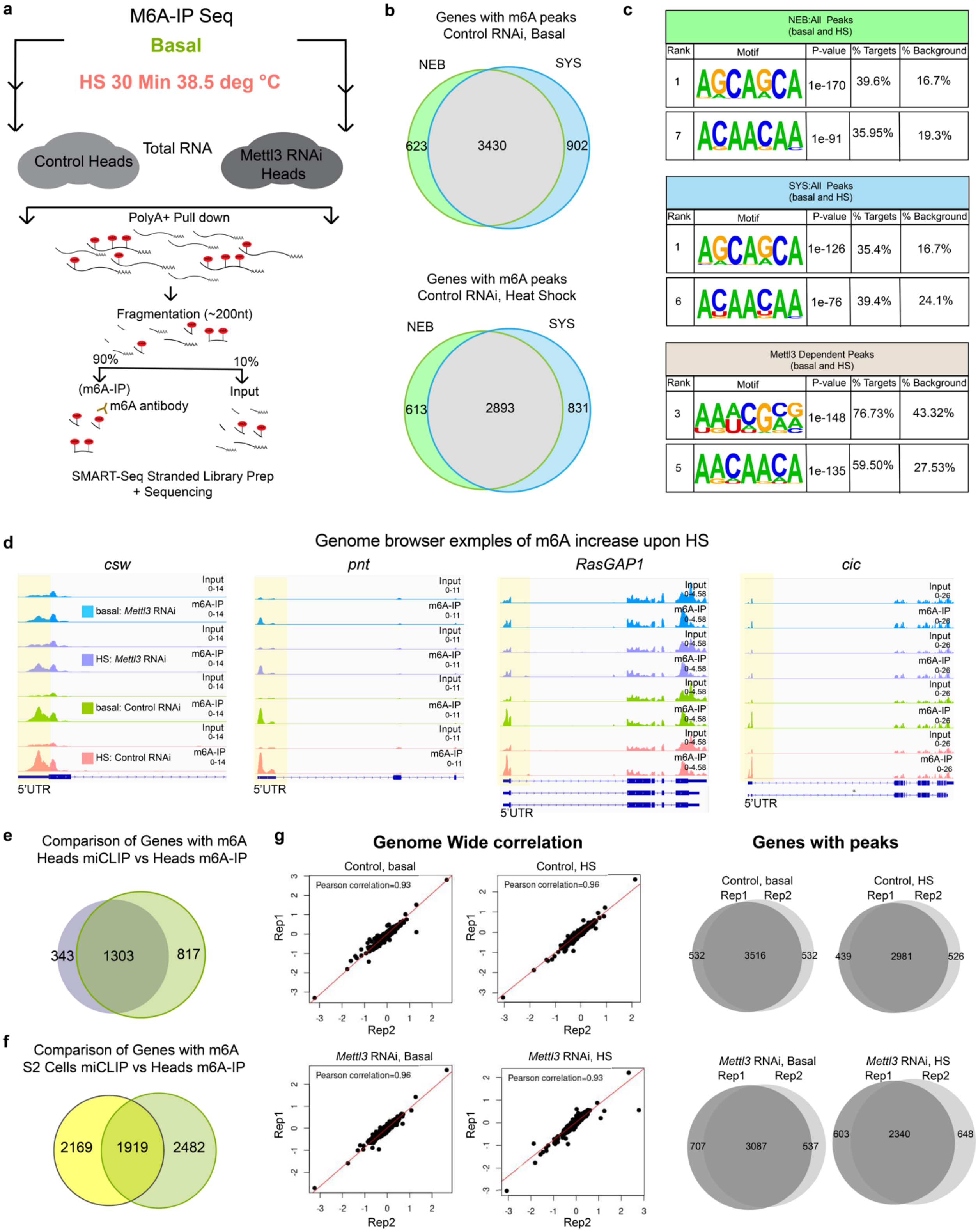

Supplementary Figure 4 m^{6}A-IP seq using two antibodies (NEB and SYS).

a Schematic diagram of the m^{6}A-IP sequencing protocol and experimental design (Genotype: DaGal4> mCherry RNAi; DaGal4> *Mettl3* RNAi).

b Overlap of genes with m^{6}A peaks from NEB and SYS m^{6}A -IP in basal conditions and HS 30 min conditions.

c De novo motif discovery from both antibody IP experiments (NEB and SYS), and from *Mettl3*-dependent m^{6}A genes. Full Motif analysis and p-values found in Supplementary Data 1.

d Example genome browser tracks of m^{6}A genes that show increase m6A upon HS: *csw, pnt, RasGAP1, cic* in basal and HS 30 min from control and *Mettl3* RNAi brains. The yellow box highlights the *Mettl3*-dependent m^{6}A peak in the 5'UTR.

f Overlap of m^{6}A peaks from head tissue m^{6}A-IP (SYS antibody, this study) with miCLIP-seq m^{6}A peaks from head tissue (SYS)[39].

g Overlap of m^{6}A-IP determined m^{6}A peaks from head tissue (SYS antibody, this study) with miCLIP-seq from S2 cells (SYS)[40].

h Genome wide correlation of log(m6A/input) read coverage comparing rep 1 to rep 2 of m^{6}A-IP seq experiments from SYS antibody. Pearson correlation shown for each comparison (left). Comparison of genes with m^{6}A peaks called from each individual replicate m^{6}A-IP seq in each condition (right). For MetPeak and RADAR peak calling both replicates are taken into account and call peaks/ differential peaks common to both reps.

Source data and statistical analysis are provided as a Source Data file.

Supplementary Figure 5 Analysis of m^{6}A genes

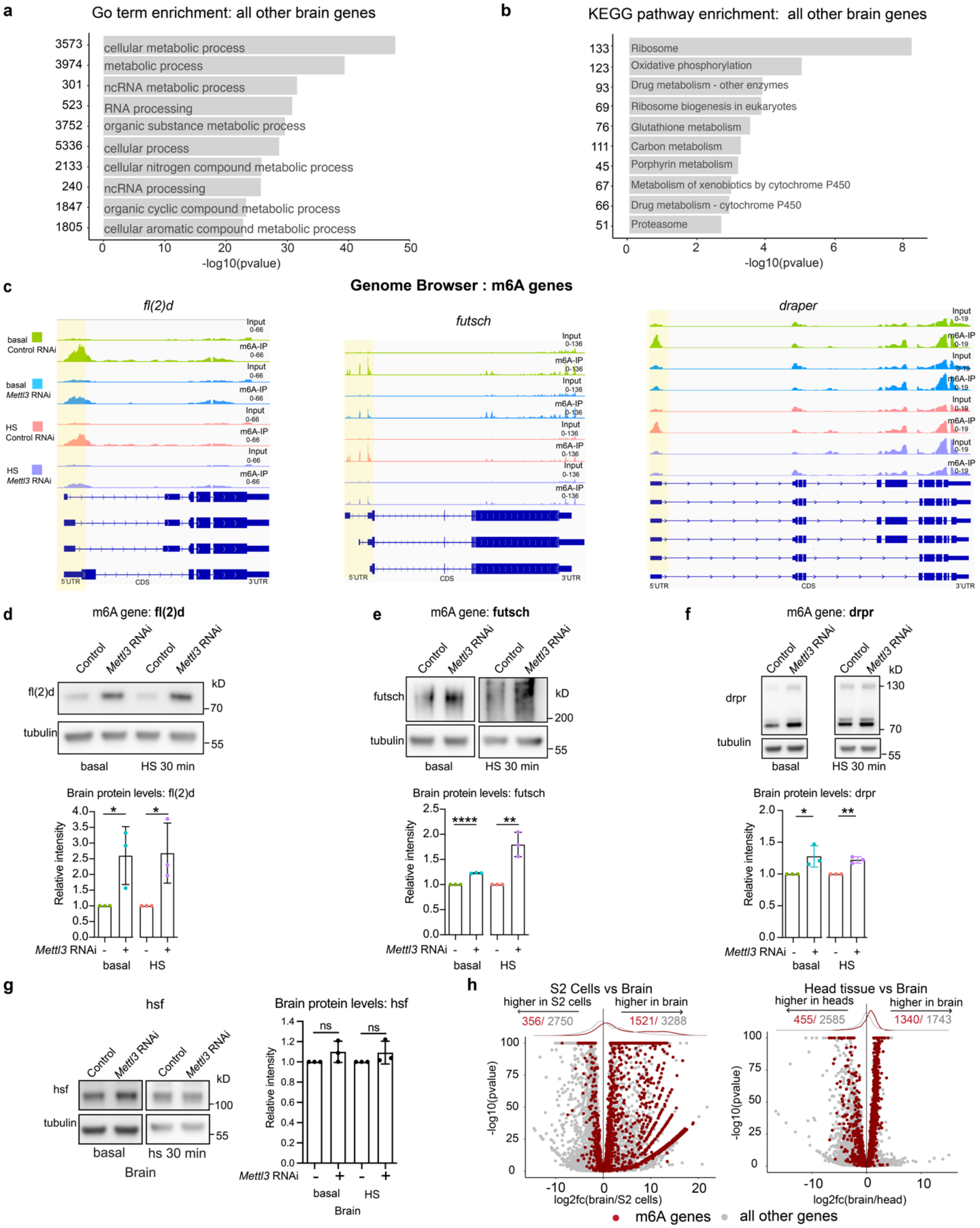

Supplementary Figure 5 Analysis of m^{6}A genes

a-b GO term and KEGG pathway enrichment of all genes expressed in the brain that are not m^{6}A modified. The -log10(pval) enrichment of genes in each category is shown, as well as the number of genes that fall into each category. Full Go-term list and p-values provided in Supplementary Data 3.

c Example genome browser tracks of m^{6}A genes *fl(2)d, futsch, and drpr* in basal and HS 30 min from DaGal4> mCherry RNAi or DaGal4> *Mettl3* RNAi brains.

d-e fl(2)d, futsch, drpr protein levels from DaGal4> mCherry RNAi or DaGal4> *Mettl3* RNAi brains dissected in basal conditions or immediately after HS 30 min at 38.5 °C. Quantification (below) of 3-biological replicate immunoblots, 15 brains per replicate, showing increased expression of protein in *Mettl3* RNAi fly brain in basal and HS. Data are presented as mean ± SD, *p<0.05, **p<0.01, ****p<0.0001, Student's two-tailed t-test. For fl(2)d, p= 0.0396, p= 0.0382, for futsch, p<0.0001, p= 0.0047, for drpr, p= 0.0454, p= 0.0016.

g Protein levels of Hsf (non m^{6}A gene) from DaGal4> mCherry RNAi or DaGal4> *Mettl3* RNAi brains dissected in basal or HS 30 min at 38.5 °C. n=3, Data are presented as mean ± SD, Student's two-tailed t-test, ns= not significant.

h Volcano plots highlighting genes with m^{6}A (red), or non-m^{6}A (grey). Plots show logFC expression of transcripts in S2 cells vs brains, or heads vs brains. Relative abundance of each colored gene set on the upper histogram. m^{6}A genes were more highly expressed in the brain. Differential expression analysis provided in Supplementary Data 4.

Source data and statistical analysis are provided as a Source Data file.

Supplementary Figure 6 RNA-sequencing from *Drosophila* brains in control and *Mettl3* RNAi in basal, HS 30 min, and recovery timepoints.

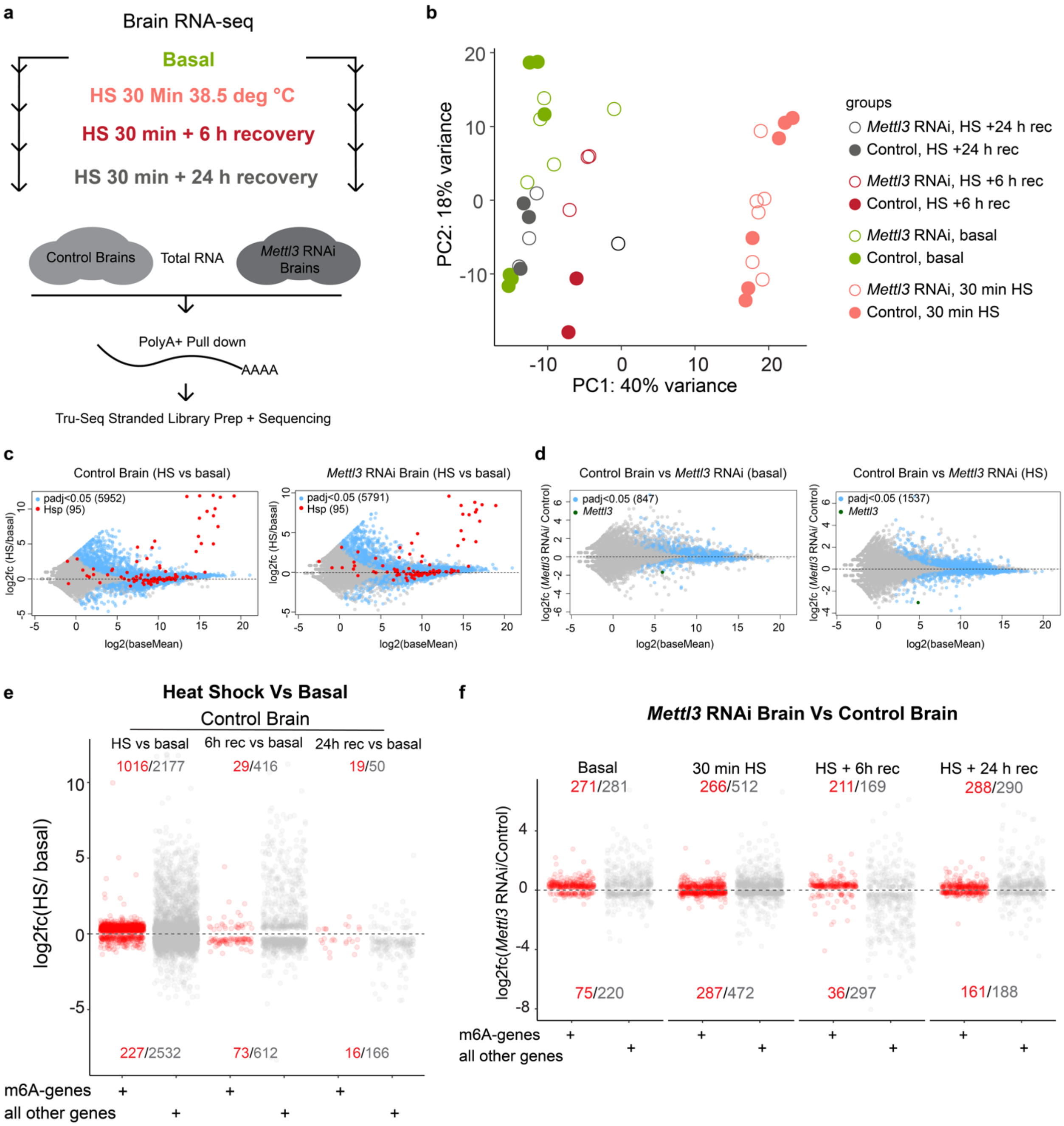

Supplementary Figure 6 RNA-sequencing from *Drosophila* brains in control and *Mettl3* RNAi in basal, HS 30 min, and recovery timepoints.

a Schematic diagram of the HS brains RNA-seq experiment. DaGal4> mCherry RNAi or DaGal4> *Mettl3* RNAi brains were dissected at basal, after HS 30min, HS + 6 h recovery, and HS + 24 h recovery. n=3 biological replicates per condition and n=20 brains per replicate.

b Principal component analysis (PCA) plot of control brains and *Mettl3* RNAi brains from basal, HS 30 min, HS + 6 h recovery, and HS + 24hr recovery RNA-sequencing. Each circle is one biological replicate.

c MA plot showing number of differentially expressed genes (blue dots, padj<0.05) and HS protein genes (red dots, sup file 1 for list of genes) from control and *Mettl3* RNAi brains, basal versus HS 30 min. Differential expression analysis provided in Supplementary Data 5.

d MA plot showing number of differentially expressed genes (blue dots, padj<0.05) and in basal and HS conditions from control brains versus *Mettl3* RNAi brains. (*Mettl3* knockdown fold change marked in dark green dot). Differential expression analysis provided in Supplementary Data 5.

e Plot of significantly differentially expressed genes in HS vs basal, 6 h recovery vs basal, and 24 h recovery vs basal for control brains. Positive logFC indicates an increase in transcript levels with HS or HS recovery. Differential expression analysis provided in Supplementary Data 5.

f Plot of significantly differentially expressed genes in *Mettl3* RNAi vs control brains, at basal, HS, 6 h recovery, and 24 h recovery conditions. Positive logFC indicates an increase in transcript levels in *Mettl3* RNAi brains. Differential expression analysis provided in Supplementary Data 5.

Source data and statistical analysis are provided as a Source Data file.

Supplementary Figure 7 M6A-IP Sequencing from *Drosophila* in control and *Mettl3* RNAi in basal and HS 30 min conditions.

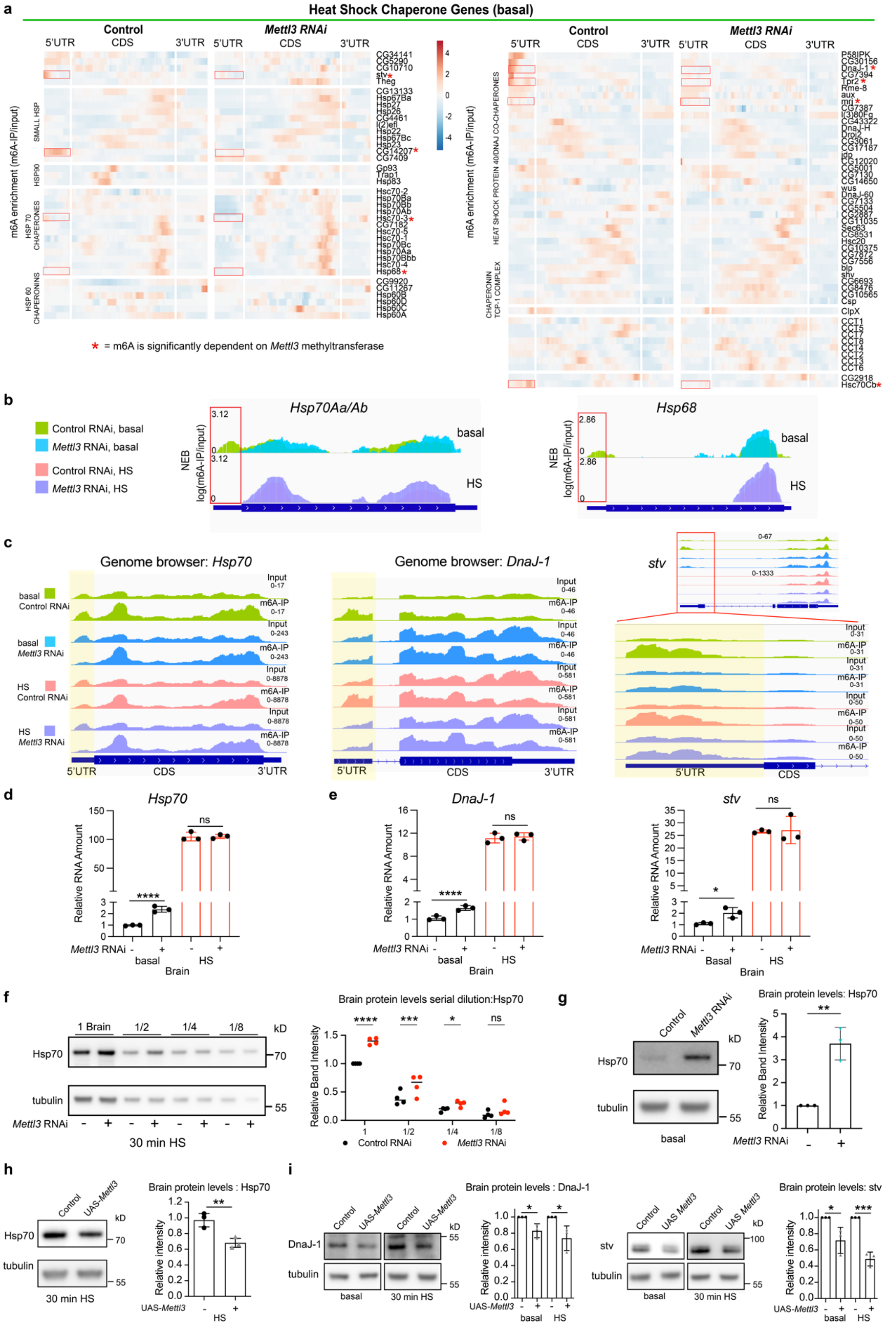

Supplementary Figure 7 M^6A-IP Sequencing from *Drosophila* in control and *Mettl3* RNAi in basal and HS 30 min conditions.

a Heat map of m^6A enrichment on HS Chaperone Genes (as defined by fly base, Supplementary Data 6) in basal conditions from DaGal4> mCherry RNAi or DaGal4> *Mettl3* RNAi heads. m^6A enrichment is shown as log (m^6A -IP divided by the input control). Heat map displays z-score values which is scaled by row, each gene is relative to itself and relative across all 6 boxes. Segmented into 5'UTR, CDS, and 3'UTR.

b Example tracks from genome browser of m^6A locations for HS chaperone transcripts *Hsp70Aa/Ab* and *Hsp68* in basal and HS 30 min from control and *Mettl3* RNAi brains with NEB m^6A antibody m^6A-IP. Tracks shown as log(m^6A-IP divided by the input control).

c Genome browser of m^6A peaks for HS chaperone transcripts *Hsp70Aa/Ab, DnaJ-1,* and *stv* in basal and HS 30 min from Control and *Mettl3* RNAi brains with SYS m^6A antibody m^6A-IP.

d-e RNA levels of *Hsp70*, *DnaJ-1, stv* in control and *Mettl3* RNAi brains in basal and HS conditions were assessed by RT-qPCR. n=3 biological replicates, 15 brains per replicate. Data are presented as mean ± SD, *p<0.05, ****p<0.0001, Student's two-tailed t-test. For *Hsp70* p= 0.0007, for *DnaJ-1* p=0.0058, for *stv* p=0.0082, ns= not significant.

f Protein levels of Hsp70 levels from control RNAi and *Mettl3* RNAi brains with 30 min HS (*DaGal4> Mettl3 RNAi vs* mCherry RNAi), and serial dilutions of brain protein samples. Quantification of 3-biological immunoblots, 15 brains per replicate. Data are presented as mean ± SD, *p<0.05, ***p<0.001, ****p<0.0001, two-way ANOVA with Sidak's test. p<0.0001, p=0.0001, p=0.0495, ns= not significant.

g Protein levels of Hsp70 levels from control RNAi and *Mettl3* RNAi brains at baseline (DaGal4> mCherry RNAi or DaGal4> *Mettl3* RNAi). Quantification of 3-biological immunoblots, 15 brains per replicate. Data are presented as mean ± SD, **p<0.01, Student's two-tailed t-test. p= 0.0028.

h Hsp70 protein levels from control and UAS-*Mettl3* upregulation brains (ElavGS> UAS-mCherry vs ElavGS> UAS-*Mettl3,* 6d on RU-486) dissected immediately after HS 30 min at 38.5 °C. n=3 biological replicates, 15 brains per replicate. Data are presented as mean ± SD, **p<0.01, Student's two-tailed t-test. p= 0.0083.

k DnaJ-1 and stv protein levels from control and UAS-*Mettl3* upregulation brains (ElavGS> UAS-mCherry vs ElavGS> UAS-*Mettl3,* 6d on RU-486) dissected in basal conditions or after HS 30 min at 38.5 °C. n=3 biological replicates, 15 brains per replicate. Data are presented as mean ± SD, *p<0.05, ***p<0.001 Student's two-tailed t-test. p= 0.0267, p= 0.0404, p= 0.0384, p= 0.0005.

Source data and statistical analysis are provided as a Source Data file.

Supplementary Figure 8 Actinomycin assay from brains

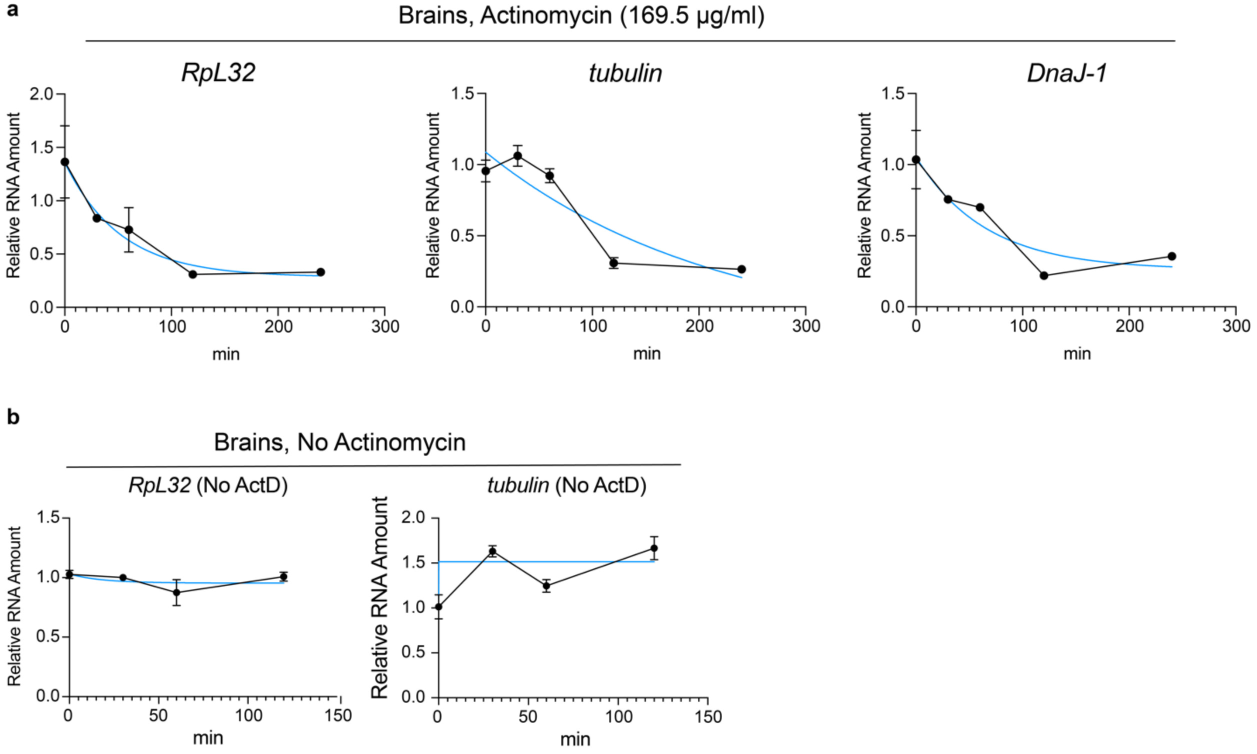

Supplementary Figure 8 Actinomycin assay from brains

a Actinomycin D assay from control (BL5905) fly brains dissected and incubated in Schneider's *Drosophila* Medium plus 169.5µg/ml of actinomycin D for 30 min, 1hr, 2hrs, 4hrs, and a no addition of actinomycin D control. RNA was extracted and used for RT-qPCR to determine relative RNA levels of *tubulin, DnaJ-1* and *RpL32*. Each data point represents, n=3 biological replicates, 20 brains per replicate. Blue line is best fit curve.

b Control (BL5905) fly brains dissected and incubated in Schneider's *Drosophila* Medium plus without actinomycin D for 0 min, 30 min, 1hr, and 2hrs. RNA was extracted and used for RT-qPCR for expression of *RpL32* and *tubulin*. Each data point represents mean, n=3 biological replicates per condition, 20 brains per replicate. Blue line is best fit curve.

Source data and statistical analysis are provided as a Source Data file.

Supplementary Figure 9 Protein translation altered with *Mettl3* knockdown.

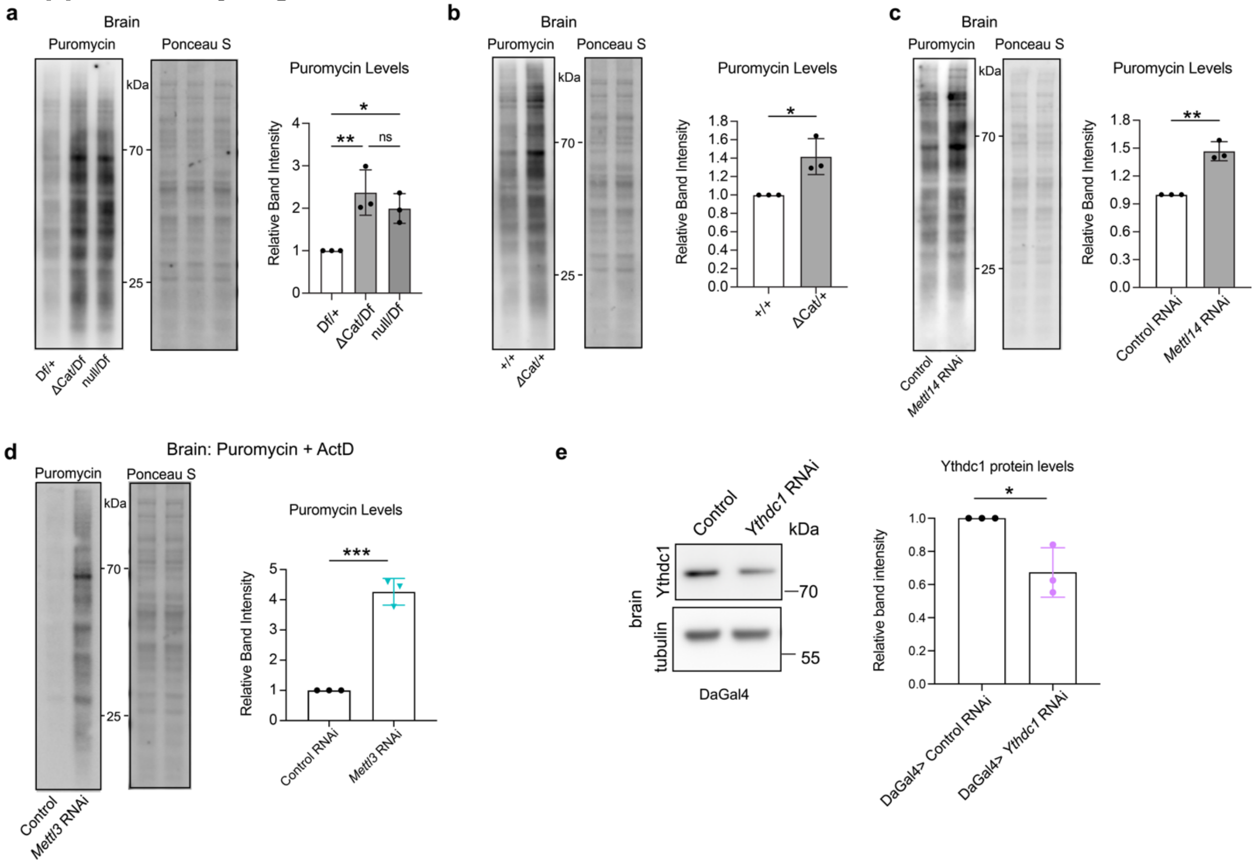

Supplementary Figure 9 Protein translation altered with *Mettl3* knockdown.

a Catalytic *Mettl3* mutant/*Mettl3* deficiency (ΔCat/Df), null *Mettl3* mutant/ *Mettl3* deficiency (Null/Df), And wildtype/ *Mettl3* deficiency (+/Df) brain puromycin assay. n=3 biological replicates, 15 brains dissected per replicate. Data are presented as mean ± SD, *p<0.05, **p<0.01, One-way ANOVA. p=0.0093, p=0.0377, ns= not significant.

b Catalytic *Mettl3* mutant/wildtype (ΔCat/+), versus wildtype (+/+) brain puromycin assay. n=3 biological replicates, 15 brains per replicate. Data are presented as mean ± SD, *p<0.05, Student's two-tailed t-test. p= 0.0209.

c Dagal4> mCherry RNAi or Dagal4>*Mettl14* RNAi brain puromycin assay. n=3 biological replicates, 15 brains per replicate. Data are presented as mean ± SD, **p<0.01, Student's two-tailed t-test. p= 0.0014.

d Dagal4> mCherry RNAi vs Dagal4>*Mettl3* RNAi brain puromycin assay + Actinomycin D treatment. Flies were fed 600uM puromycin + Actinomycin D (transcription inhibitor) for 24 hours. n=3 biological replicates, 15 brains per replicate. Data are presented as mean ± SD, **p<0.01, Student's two-tailed t-test. p= 0.0002.

e Dagal4>mCherry RNAi vs Dagal4>*Ythdc1* RNAi brains probed for Ythdc1 protein levels. n=3 biological replicates, 15 brains per replicate. Data are presented as mean ± SD, *p<0.05, Student’s two-tailed t-test. p= 0.0192.

Source data and statistical analysis are provided as a Source Data file.
