# Supplementary material for: Mettl3-dependent m6A modification attenuates the brain stress response in Drosophila
Source: Nat Commun. 2022 Sep 14;13:5387. doi: 10.1038/s41467-022-33085-3 (PMC9474545; doi:10.1038/s41467-022-33085-3)
Supplement: Supplementary file 3 — Description of Additional Supplementary Files [file 41467_2022_33085_MOESM3_ESM.pdf]

Description of Additional Supplementary Files:

Supplementary Data 1: Full De-novo Motif files

Sheet 1, Motifs From SYS.
Sheet 2, Motifs from NEB.
Sheet 3, Motifs from *Mettl3* dependent genes

Supplementary Data 2: m^6A Gene Lists.RADAR.

Sheet 1, *Mettl3*-Dependent m6A peak Genes.
Sheet 2, Radar: *Mettl3* RNAi vs mCherry RNAi Synaptic systems antibody basal conditions.
Sheet 3, Radar: *Mettl3* RNAi vs mCherry RNAi Synaptic systems antibody HS 30 min conditions.
Sheet 4, Radar: Basal vs HS in Control (mCherry RNAi) Synaptic systems antibody HS 30 min conditions.

Supplementary Data 3: GO term and Kegg Pathway lists

List of all go terms:
Sheet 1, *Mettl3* Dependent GO terms.
Sheet 2, *Mettl3* Dependent Kegg Terms.
Sheet 3, non-m6A Brain Genes GO terms.
Sheet 4, non-m6A Brain Genes Kegg.
Sheet 5, *Ythdc* RNAi Go terms all upregulated genes.
Sheet 6, m6A tagged genes Upregulated with Ythdc RNAi

Supplementary Data 4: S2 cell vs brain, head vs brain RNA-seq

Drosophila tissue types, RNA-seq differential gene expression comparison table. Comparison of Brain versus S2 cells. Comparison of Brain versus total head tissue

Supplementary Data 5: Brain RNA-seq Differential expression lists

Brain Differential expression lists. RNA sequencing timecourse in brains from control, *Mettl3* RNAi, *Ythdc* RNAi *Drosophila* brains, basal and HS conditions)

Supplementary Data 6: FlyBase HS gene list

List of HSP chaperones defined by fly base (https://flybase.org/reports/FBgg0000501.html). Added Top 20 significantly differentially expressed genes with HS from our Brain-RNA-seq (*any not present original fly base list). Additional information from brain RNA-seq on LogFC levels with HS in the brain, padj, direction change with HS.

Supplementary Data 7: *Drosophila* Lines and Primers used

List of *Drosophila* lines used in the paper and *Drosophila* crosses used in each figure. List of Primers used for RT-qPCR

Supplementary Data 8: Mapping rates for all sequencing experiments

M6A-IP and RNA-seq mapped reads table and description of experiment and biological replicates.
e1= m6A in basal and heat shock heads - m6A-IP pulldown with SYS antibody.
e2 = m6A in basal and heat shock heads -m6A-IP pulldown with NEB antibody.
e3= RNA-seq timecourse of heat shock (basal, heat shock, heat shock + 6 hr recovery, heat shock +24 hr recovery). e7= RNA-seq of heat shock with *Ythdc* knockdown.
