# Supplementary material for: Mettl3-dependent m6A modification attenuates the brain stress response in Drosophila
Source: Nat Commun. 2022 Sep 14;13:5387. doi: 10.1038/s41467-022-33085-3 (PMC9474545; doi:10.1038/s41467-022-33085-3)
Supplement: Supplementary file 12 — Reporting Summary [file 41467_2022_33085_MOESM12_ESM.pdf]

nature portfolio

Corresponding author(s): Nancy M. Bonini

Last updated by author(s): Aug 22, 2022

Reporting Summary

Nature Portfolio wishes to improve the reproducibility of the work that we publish. This form provides structure for consistency and transparency in reporting. For further information on Nature Portfolio policies, see our Editorial Policies and the Editorial Policy Checklist.

Statistics

For all statistical analyses, confirm that the following items are present in the figure legend, table legend, main text, or Methods section.

n/a	Confirmed	
☐	☒	The exact sample size (*n*) for each experimental group/condition, given as a discrete number and unit of measurement
☐	☒	A statement on whether measurements were taken from distinct samples or whether the same sample was measured repeatedly
☐	☒	The statistical test(s) used AND whether they are one- or two-sided<br>*Only common tests should be described solely by name; describe more complex techniques in the Methods section.*
☐	☒	A description of all covariates tested
☐	☒	A description of any assumptions or corrections, such as tests of normality and adjustment for multiple comparisons
☐	☒	A full description of the statistical parameters including central tendency (e.g. means) or other basic estimates (e.g. regression coefficient) AND variation (e.g. standard deviation) or associated estimates of uncertainty (e.g. confidence intervals)
☐	☒	For null hypothesis testing, the test statistic (e.g. *F*, *t*, *r*) with confidence intervals, effect sizes, degrees of freedom and *P* value noted<br>*Give P values as exact values whenever suitable.*
☒	☐	For Bayesian analysis, information on the choice of priors and Markov chain Monte Carlo settings
☒	☐	For hierarchical and complex designs, identification of the appropriate level for tests and full reporting of outcomes
☐	☒	Estimates of effect sizes (e.g. Cohen's *d*, Pearson's *r*), indicating how they were calculated

Our web collection on statistics for biologists contains articles on many of the points above.

Software and code

Policy information about availability of computer code

Data collection: No custom software or analyses were used. See methods section for full description of packages or code implemented.

Data analysis:

M6A enrichment analysis
Regions of m6A enrichment were found for each condition using MetPeak (v.1.1)63 with default parameters, using the input and m6A pulldown bam files as input, and with the FlyBase FB2019_05 annotation provided. Peak locations (5' UTR, CDS, or 3' UTR) were defined from the regions indicated by MetPeak as having significant m6A enrichment. If a peak was not contained in one region (i.e., if the peak is partly in the CDS and partly in the 3' UTR), it was assigned to the region where more of the peak resided.

Differential m6A peak analysis
Regions of differential methylation between two conditions (frequently called "Mettl3-dependent genes" or "m6A genes") were found using RADAR (v.0.2.4) 47 with input and m6A pulldown bam files as input, as well as the FlyBase FB2019_05 annotation. All replicates of SYS antibody used for differential peak calling. The minimum cutoff for bin filtering was 15, the cutoff was set as 0.05, and the Beta_cutoff was set as 0.5. Any region with an adjusted p-value < 0.05 was retained, and regions with a fold-change < -1 from the control (mCherry) to the knockout (Mettl3) at basal or heat shock (30 minutes) conditions were kept as dependent peaks. Non-Mettl3-dependent m6A were considered as all other genes expressed in the brain that did not have Mettl3-dependent m6A.

M6A metaplots, heatmaps, and genome browser visualization
Heatmaps and metagene plots showing the location of m6A enrichment on a specific set of genes were constructed with using pheatmaps (v.1.0.12) and meRIPtools (v.0.2.1). Specifically, the exons of all transcripts in each gene were collapsed using the GenomicRanges (v.1.44.0) function reduce 64. Genes with a 5' UTR or 3' UTR shorter than 30 base pairs, a CDS shorter than 100 bp, or lacking a 5'/3' UTR (i.e., lncRNAs)

were not considered in this analysis. For each gene, the 5' UTR and 3' UTR were tiled in 30 evenly spaced bins, and the CDS was tiled in 100 evenly spaced bins. The number of input and m6A reads overlapping each bin was calculated and this number was divided by the bin width and library size and a normalization factor of one million to produce a normalized reads per million in each bin. The heatmaps show the enrichment of m6A above input in each bin by dividing the m6A coverage by the input coverage after adding replicates from the same condition and sample type together. For heat map normalization, reads per million are normalized by the size of the bin, total reads, and library size. For genome browser snapshots, tracks visualized are log2(m6A /input), or separated input and m6A-IP tracks in supplementary figures. Tracks were made by first converting bam files to bigWig files using deepTools (v.3.5.1) 65 bamCoverage using CPM normalization, then deepTools bigwigCompare with operation log2.

GO and pathway analysis
GO analysis for genes with non-Mettl3-dependent m6A or Mettl3-dependent m6A was conducted using FlyMine (v.53) 66. The test correction was set to Holm-Bonferroni with a max p-value of 0.05. KEGG pathway analysis was done using the "enrichKEGG" function from ClusterProfiler (v.4.0.5) package in R 67. A list of all genes with detectable expression was used as background for both GO and pathway analysis.

Motif analysis
Motif enrichment for m6A obtained with pulldowns using either NEB or SYS antibodies was performed using HOMER (v.4.11) 68 findMotifs.pl with the parameters -rna -len 5,6,7. FASTA files containing the sequence of the RNA base pairs under each peak were compared with FASTA comprising of background sequences, which were generated by taking random regions of expressed transcripts without an m6A peak that were length-matched to the peak sequences.

RNA sequencing
Total RNA was extracted from brains using trizol/chloroform and Zymo RNA clean and concentrator kit-5 (R1015). The RNA-seq libraries from brains were prepared using the Tru-seq stranded mRNA library prep. Library preparation and sequencing was done by Admera Health, and sequenced using illumina NovaSeq S4 with 40M paired end reads (2x150bp). Three biological replicates were done for each experimental timepoint, condition, and genotype. Technical repeats and at least n=3-6 biological replicates of at least 15 brains per replicate were done as indicated in Supplementary Data 8.

RNA-seq analysis
Raw paired-end fastqs were processed with TrimGalore (v.0.6.6) (https://github.com/FelixKrueger/TrimGalore) with default settings to remove Illumina adapters and mapped using STAR 2.7.3a 69 to the Drosophila melanogaster genome annotation dm6. Unmapped and improperly paired reads were filtered out of aligned bam files. Reads per gene in the FlyBase release 2019_05 were computed using an R script using GenomicRanges (v.1.44.0) 70 summarizeOverlaps that counts the number of reads overlapping with the exons of each gene in the default "union" mode. Differential expression analysis was performed using DESeq2 (v.1.32.0) 71, with count files produced by summarizeOverlaps as input. PCA plots were made using the plotPCA function in DESeq2, with variance stabilized counts as the input. MA plots were constructed from the adjusted p-values and baseMean values output from DESeq2, and volcano plots were constructed from adjusted p-values and fold changes reported by DESeq2. Normalized counts produced by DESeq2 were used to show expression levels. Differentially expressed genes were considered to be any gene with a p-adjusted values of < 0.05.

Quantification and statistical analysis:
Statistical tests used were performed on GraphPad Prism (v.9), and are indicated in the figure legend. P-values of <0.05 were considered significant. Unpaired two-tailed t-tests were used when comparing 2 groups; One-way ANOVA was used when comparing multiple groups followed by Tukey's post-test when each group was compared against every other group, Sidak's post-test when pre-defined groups were compared to each other, or Dunnett's test when comparing to a defined control sample. Two-way ANOVA was used when there were 2 factors in the analysis (usually RNA amount and HS condition). Fisher's exact test was used when comparing m6A vs non m6A genes (Fig 4). One-sided Hypergeometric test was used to compare Venn diagram overlaps.

For manuscripts utilizing custom algorithms or software that are central to the research but not yet described in published literature, software must be made available to editors and reviewers. We strongly encourage code deposition in a community repository (e.g. GitHub). See the Nature Portfolio guidelines for submitting code & software for further information.

Data

Policy information about availability of data

All manuscripts must include a data availability statement. This statement should provide the following information, where applicable:

- Accession codes, unique identifiers, or web links for publicly available datasets
- A description of any restrictions on data availability
- For clinical datasets or third party data, please ensure that the statement adheres to our policy

The Raw sequencing data generated in this study have been deposited in the Gene Expression Omnibus under accession code GSE178955 [https://www.ncbi.nlm.nih.gov/geo/query/acc.cgi?acc=GSE178955]. Full Motif Analysis are available in Supplementary Data 1. The m6A peak RADAR data used in this study are available in the Supplementary Data 2. Go-term and Kegg pathway analysis are available in Supplementary Data 3. The Differential gene expression tables from RNA-seq analysis generated in this study are provided in the Supplementary Data 4 and 5. HS gene lists are provided in Supplementary Data 6. Fly genotypes and primers used are provided in Supplementary Data 7. Mapping rates for all sequencing experiments are provided in Supplementary Data 8. Any additional inquiries can be directed to the corresponding author. Source data are provided with this paper.

Human research participants

Policy information about studies involving human research participants and Sex and Gender in Research.

Reporting on sex and gender	N/A
Population characteristics	N/A
Recruitment	N/A
Ethics oversight	N/A

Note that full information on the approval of the study protocol must also be provided in the manuscript.

Field-specific reporting

Please select the one below that is the best fit for your research. If you are not sure, read the appropriate sections before making your selection.

☒ Life sciences ☐ Behavioural & social sciences ☐ Ecological, evolutionary & environmental sciences

For a reference copy of the document with all sections, see nature.com/documents/nr-reporting-summary-flat.pdf

Life sciences study design

All studies must disclose on these points even when the disclosure is negative.

Sample size	Sample size was determined based on precedent by our lab and is standard in the field. DOI: 10.1038/s41593-019-0396-1, DOI: 10.1111/acel.13559, https://doi.org/10.1038/s43587-021-00072-0.
Data exclusions	No data were excluded.
Replication	All experiments were repeated at least 2-4 times with similar results.
Randomization	Male sibling flies were randomly assigned to experimental conditions. For experiments with non-sibling flies (i.e: different genetic background), flies were age-matched and handled in parallel. Covariates were controlled through repeating experiments 3+ times from biologically distinct replicates animals (same genetic background but crosses set up from different parent flies).
Blinding	Animal crosses are given unique number identifiers. All animal tissue collected samples (protein, RNA, sequencing, etc.) were given unique sample ID's so that data acquisition and quantification was always performed blind to sample identity.

Reporting for specific materials, systems and methods

We require information from authors about some types of materials, experimental systems and methods used in many studies. Here, indicate whether each material, system or method listed is relevant to your study. If you are not sure if a list item applies to your research, read the appropriate section before selecting a response.

Materials & experimental systems

n/a	Involved in the study	
☐	☒	Antibodies
☒	☐	Eukaryotic cell lines
☒	☐	Palaeontology and archaeology
☐	☒	Animals and other organisms
☒	☐	Clinical data
☒	☐	Dual use research of concern

Methods

n/a	Involved in the study	
☒	☐	ChIP-seq
☒	☐	Flow cytometry
☒	☐	MRI-based neuroimaging

Antibodies

Antibodies used	Primary antibodies (concentration used, supplier, catalog number, lot number):<br>mouse anti-tubulin (1:5,000, DHSB, AA4.3, 5/31/18-44ug/ml)<br>rat anti-Hsp70 (1:5,000, Sigma, 7FB-SAB5200204-100uG,141002)<br>rabbit anti-Mettl3 (1:5,000, Proteintech, 15073-1-AP, Ag7110)

	rabbit anti-HSP40 (1:5,000, Enzo Life Sciences, ADI-SPA-400-D,04062141)<br>rabbit anti-stv (1:5,000, Proteintech, 13913-1-AP, Ag4905)<br>mouse anti-fl(2)d (1:10, DSHB-9G2, 10/18/18-42ug/ml)<br>mouse anti-futsch (1:600, DSHB-22C10, 10/10/19-53ug/ml)<br>mouse anti-drpr (1:400, DSHB-5D14, 6/22/17 -36ug/ul)<br>mouse anti-puromycin (1:1,000, Kerafast, EQ0001, 200517)<br>mouse anti-hsf (1:20:000, anti-rabbit, gift from John Lis, DOI: 10.1038/353822a0)<br>rabbit anti-m6A (1:1,000, Synaptic systems,202003, 2-97)<br>rabbit anti-m6A(NEB, E1611A, 10015190)<br>rabbit Anti-Ythdc1(1:5,000, Generated by lab at Vivitide)<br><br>Secondary antibodies (concentration used, supplier, catalog number, lot number):<br>Goat anti-rabbit HRP (1:5,000, Jackson ImmunoResearch, 111-035-144, 138306)<br>Goat anti-mouse IgG H&L HRP (1:5,000, Jackson ImmunoResearch, 115-035-146,153978)<br>Goat anti-rat (1:5,000, Thermo Fisher Scientific, A10549, 2273679)<br><br>Methods details specific concentrations used for different applications.
Validation	Primary antibodies (concentration used, supplier, catalog number, lot number):<br>anti-tubulin: well-established, correct band size, see https://dshb.biology.uiowa.edu/AA4-3<br>anti-Hsp70: confirmed in Drosophila, https://www.sigmaaldrich.com/US/en/product/sigma/sab5200204<br>anti-Mettl3: validated using knockdown of Mettl3 in this paper, manufacturer references use in Drosophila, https://www.ptglab.com/products/METTL3-Antibody-15073-1-AP.htm#product-information<br>anti-HSP40: band at expected molecular weight, increases with heat stress as expected, cross-reactivity with many species due to homology of protein, https://www.enzolifesciences.com/ADI-SPA-400/hsp40-hdj1-polyclonal-antibody/, https://antibodyregistry.org/search.php?q=AB_2039237<br>anti-stv: Validated in this paper through knockdown of STV RNAi, RNAi validated through RT-qPCR, single band seen, See source data file, protein becomes up-regulated with stress as expected in whole fly tissue.<br>anti-fl(2)d: confirmed in Drosophila, https://dshb.biology.uiowa.edu/22C10<br>anti-futsch: confirmed in Drosophila, https://dshb.biology.uiowa.edu/Fl-2-d-9G2<br>anti-drpr: confirmed in Drosophila, https://dshb.biology.uiowa.edu/Draper-5D14<br>anti-puromycin: antibody directed toward puromycin drug, several studies using this antibody in Drosophila https://www.kerafast.com/productgroup/190/anti-puromycin-3rh11-antibody<br>anti-hsf: well established in drosophila, DOI: 10.1038/353822a0<br>anti-m6A: against m6A modified RNA , used in most m6A-IP studies, https://www.sysy.com/product/202003<br>anti-m6A: against m6A modified RNA , used in several m6A-IP studies https://doi.org/10.1016/j.cell.2017.03.031.<br>anti-Ythdc1: Generated by lab and validated in source data file and supplemental data 9e. Affinity purified rabbit antibody created by Vivitide against 18 residues of Ythdc1 (157-173 "CRTKIPSNANDSAGHKSD"). Single band at expected molecular weight seen.

Animals and other research organisms

Policy information about studies involving animals; ARRIVE guidelines recommended for reporting animal research, and Sex and Gender in Research

Laboratory animals	Male Drosophila melanogaster were used for this study. Animal age varied by experiment. For all HS experiments, 6d old males. See Supplementary Data 7 for detailed genotype information.
Wild animals	This study did not involve wild animals.
Reporting on sex	This study used male Drosophila.
Field-collected samples	This study did not involve field-collected samples.
Ethics oversight	All live animals were invertebrates, ethical approval and oversight are not required.

Note that full information on the approval of the study protocol must also be provided in the manuscript.
